# Hydrostatic Pressure Does Not Cause Detectable Changes in Survival of Human Retinal Ganglion Cells

**DOI:** 10.1371/journal.pone.0115591

**Published:** 2015-01-30

**Authors:** Andrew Osborne, Amal Aldarwesh, Jeremy D. Rhodes, David C. Broadway, Claire Everitt, Julie Sanderson

**Affiliations:** 1 School of Pharmacy, University of East Anglia, Norwich, United Kingdom; 2 School of Biological Sciences, University of East Anglia, Norwich, United Kingdom; 3 Department of Ophthalmology, Norfolk and Norwich University Hospital, Norwich, United Kingdom; 4 Pfizer Ltd, Design Centre of Excellence, Granta Park, Great Abington, Cambridge, United Kingdom; Hanson Institute, AUSTRALIA

## Abstract

**Purpose:**

Elevated intraocular pressure (IOP) is a major risk factor for glaucoma. One consequence of raised IOP is that ocular tissues are subjected to increased hydrostatic pressure (HP). The effect of raised HP on stress pathway signaling and retinal ganglion cell (RGC) survival in the human retina was investigated.

**Methods:**

A chamber was designed to expose cells to increased HP (constant and fluctuating). Accurate pressure control (10-100mmHg) was achieved using mass flow controllers. Human organotypic retinal cultures (HORCs) from donor eyes (<24h *post mortem*) were cultured in serum-free DMEM/HamF12. Increased HP was compared to simulated ischemia (oxygen glucose deprivation, OGD). Cell death and apoptosis were measured by LDH and TUNEL assays, RGC marker expression by qRT-PCR (*THY-1*) and RGC number by immunohistochemistry (NeuN). Activated p38 and JNK were detected by Western blot.

**Results:**

Exposure of HORCs to constant (60mmHg) or fluctuating (10-100mmHg; 1 cycle/min) pressure for 24 or 48h caused no loss of structural integrity, LDH release, decrease in RGC marker expression (*THY-1*) or loss of RGCs compared with controls. In addition, there was no increase in TUNEL-positive NeuN-labelled cells at either time-point indicating no increase in apoptosis of RGCs. OGD increased apoptosis, reduced RGC marker expression and RGC number and caused elevated LDH release at 24h. p38 and JNK phosphorylation remained unchanged in HORCs exposed to fluctuating pressure (10-100mmHg; 1 cycle/min) for 15, 30, 60 and 90min durations, whereas OGD (3h) increased activation of p38 and JNK, remaining elevated for 90min post-OGD.

**Conclusions:**

Directly applied HP had no detectable impact on RGC survival and stress-signalling in HORCs. Simulated ischemia, however, activated stress pathways and caused RGC death. These results show that direct HP does not cause degeneration of RGCs in the *ex vivo* human retina.

## Introduction

Glaucoma is a group of optic neuropathies leading to progressive loss of visual field due to the degeneration of retinal ganglion cells (RGCs) in the inner retina and loss of their axons in the optic nerve [1]. Vision loss caused by glaucoma is irreversible. Glaucoma is the second most common cause of world blindness after cataract [2] and thus the most common cause of irreversible blindness. Raised intraocular pressure (IOP) is a major risk factor for glaucoma [1, 3] and current glaucoma management is aimed at reducing IOP to limit neuronal damage. IOP above the normal range of 11 to 21mmHg has been shown to increase the likelihood of developing glaucoma with higher pressures leading to a progressive worsening of vision [4–7]. Fundamental questions remain, however, as to the mechanism by which elevated IOP causes degeneration of the RGCs and subsequent loss of vision in glaucoma [8].

It has proven difficult to isolate the contribution of individual variables that are affected in the eye as a result of increased IOP, which may subsequently lead to RGC death. One direct component affected by raised IOP is an increase in hydrostatic pressure (HP): when IOP increases in the eye, the retina will experience an increase in HP, acting transversely across the retina. *In vitro* studies, modelling this increase, have suggested exposing RGCs to raised HP may have a direct effect on survival [9–12], further suggesting that HP has a role in RGC death in glaucoma. Changes in cell survival have been detected in isolated RGCs exposed to short term pressure elevations of 50–70 mmHg [9, 10, 13]. Effects of HP elevations have not been investigated using human *in vitro* retinal models. The aim of the present study was to identify whether increased HP had a direct effect on cell survival in human RGCs. To achieve this aim a pressure chamber was designed and constructed and the effect of raised HP was investigated using human organotypic retinal culture (HORC) used to model retinal disease in our lab [14, 15]. The chamber was designed to limit possible confounding factors such as mechanical distortion of the tissue or fluid currents. The use of explant cultures permits examination in a directly *ex vivo* situation in which retinal cells maintain microarchitecture and cell-to-cell communication. Additionally, signalling pathways associated with stress were investigated in response to increased HP.

## Materials and Methods

### Human Organotypic Retinal Cultures (HORCs)

Donor human eyes were obtained from the East Anglian Eye Bank with ethical approval (Ref 04/Q0102/57; NHS Research Ethics Committee), with written consent from the donors’ next-of-kin and in compliance with the tenets of the Declaration of Helsinki. Retinal dissection and HORC preparation was performed as described previously [14]. Briefly, the retina was separated from the globe and dissected to give a flat retinal preparation. Five para-macular retinal explants were taken from each donor eye using a 4mm diameter, dissecting trephine (Biomedical Research Instruments, MD, USA). HORC explants were transferred to serum-free (SF) Dulbecco’s Modified Eagle Medium (DMEM)/HamF12 (Invitrogen, Paisley, UK) containing 50μg/ml gentamicin (Sigma-Aldrich, Poole, UK) in a 35mm culture dish (Corning, NY, USA). Individual HORCs were transferred to separate culture dishes containing fresh medium and incubated for 1h in a humidified atmosphere of 95% Air/5% CO_2_ prior to experimentation. Throughout the experimental period, the explants were contained in 35mm dishes containing 1.5ml SF DMEM/HamF12. The explants were submerged in the medium, but not in contact with the base of the dish. Only eyes within 24h *post mortem* were used for research and those with known/evident retinal disease such as glaucoma, age-related macular degeneration or diabetic retinopathy were excluded. In total 68 human eyes, from donors aged 43 to 84 years, were used in the experiments.

### Pressure System

**Figure 1:**
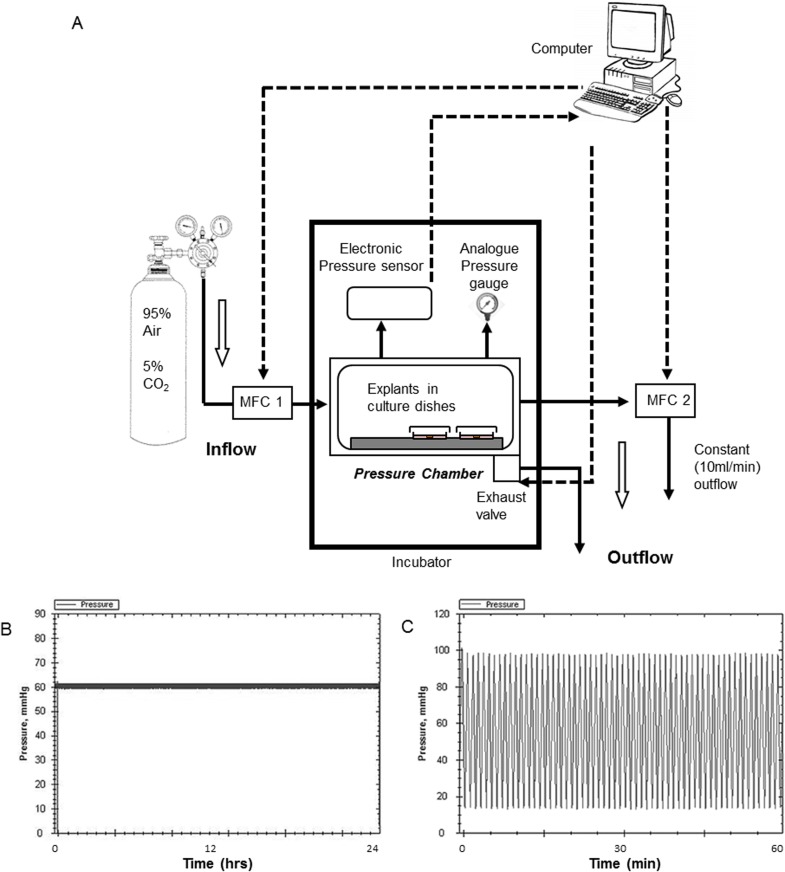
The system used to expose retinal tissue to raised hydrostatic pressure. (A) Schematic diagram of the hydrostatic pressure system (not to scale). Examples of computer controlled protocols using the pressure system at (B) constant (60mmHg) pressure for 24h and (C) fluctuating (10–100mmHg; 1 cycle/min) pressure for 1h. MFC = mass flow controller.

A custom-made chamber was constructed (UEA mechanical workshop, Norwich, UK) from Perspex to expose tissue explants to increased HP (Fig. 1A). Chamber internal dimensions were 260mm x 130mm x 140mm giving an overall volume within the chamber of 4732ml. A Perspex door was used to seal the chamber against a continuous rubber O-ring. Explants were placed inside the chamber on a raised platform in 35mm culture dishes. The dishes had lids, which were loosely fitted allowing gas exchange and equilibration of pressure. The base of the chamber was flooded with sterile deionised water in order to maintain humidity.

The chamber used mass flow controllers (MFCs), positioned at the inlet and outlet ports, to simultaneously regulate the internal pressure and the rate of gas flow through the chamber. Pressurised gas (95% air/ 5% CO_2_) could be rapidly injected into the chamber using a 1000ml/min MFC and released via a solenoid exhaust valve. Custom written software regulated internal pressure based on levels measured by a digital pressure sensor (Omega Engineering Inc, Manchester, UK). The software was able to control gas flow via an analogue to digital interface which operated the MFC and exhaust valve (Fig. 1B). The time required for compression between 10 and 100mmHg was approximately 30 seconds. The chamber regulated to ±1mmHg around the selected set-point (therefore at “constant” 60mmHg, the pressure varied between 59 and 61mmHg). Fig. 1B shows a constant pressure trace (HP(C); 60mmHg for 24h); Fig. 1C shows a fluctuating pressure trace (HP(F): 10–100mmHg; 1 cycle/min for 60 min). A second low capacity (100ml/min) MFC positioned on the outflow ensured a constant flow of gas through the chamber at 10ml/min that was independent of pressure. In order to give an analogue readout, a manometer was also fitted to the chamber. No compensation for changes in atmospheric pressure were made: the raised HP in the chamber was in addition to atmospheric pressure. Controls were maintained at atmospheric pressure in the same incubator.

No significant changes in pH or evaporation rate were detected between control and medium exposed to pressure for the experimental period (data not shown). pH was measured following removal of the medium from the chamber using a glass electrode (ThermoScientific, Loughborough, UK). Evaporation was assessed by weighing the medium before and after exposure to experimental conditions.

### Measurement of dissolved oxygen concentration

**Figure 2:**
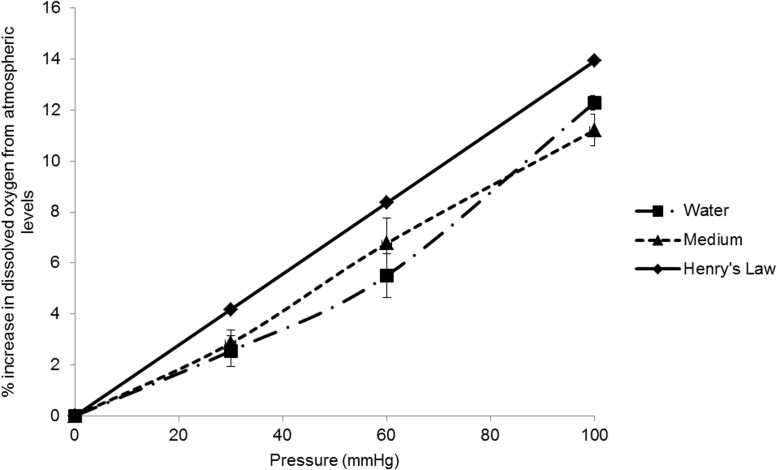
Changes in dissolved O_2_ with increased HP above atmospheric pressure. O_2_ concentration in water and medium is expressed as the percentage of the concentration recorded at atmospheric pressure (n = 4). The gas in the chamber was 95% air/ 5% CO_2_. The O_2_ concentration in pure water predicted by Henry’s Law is also shown.

O_2_ concentration in distilled water or culture medium exposed to pressure was measured using a Hansatech DW1 Oxygen Electrode (Hansatech Instruments Ltd, Norfolk, UK). The system was calibrated before each use with air saturated water or medium and oxygen-free water or medium (bubbled with 95% N_2_, 5% CO_2_ for 10min). 35mm culture dishes containing 1.5ml solution were exposed to various pressures or control conditions for 30min. 1ml of treated solution was then placed in the oxygen electrode reaction vessel. Oxygen concentrations were measured every second for ~1min whilst constantly stirring at 450rpm. The mean values for each oxygen concentration measurement were recorded (nmol/ml). The effect of pressure on O_2_ concentration in our pressure system closely followed that predicted by Henry’s Law [16] where the amount of a given gas that dissolves in a liquid is directly proportional to the partial pressure of that gas in equilibrium with the liquid (Fig. 2). The deviation from Henry’s Law likely reflects oxygen loss in the time taken between sampling and measurement. Correlation between predicted and measured O_2_ concentration further validates that the pressure in the chamber was at the designated set pressure.

### Simulated ischemia

HORCs were exposed to oxygen glucose deprivation (OGD) as described previously [14]. Briefly, 1h following dissection, the medium was changed to glucose-free DMEM. Explants were then placed in a modular incubator chamber (Billups-Rothenburg, CA, USA) gassed with 95% N_2_/5% CO_2_ and placed in an incubator at 35°C for 3h. Control cultures underwent the same number of medium changes except using DMEM (containing glucose) and were incubated at atmospheric conditions in the same incubator as the modular chamber. Samples were directly processed, or medium was exchanged for SF DMEM/HamF12 (containing glucose) until the experimental end point.

### Lactate dehydrogenase (LDH) assay

The level of cell death was determined by measuring the LDH activity in cell culture medium according to the manufacturer’s instructions (Roche Molecular Biochemicals, Burgess Hill, UK).

### Quantitative Real Time PCR (qRT-PCR)

Total RNA was extracted from HORCs using the RNeasy Mini Kit (Qiagen, Crawley, UK) according to the manufacturer’s instructions. The concentration of total RNA was measured using a NanoDrop ND-1000 spectrophotometer (NanoDrop Technologies, Wilmington, USA). Total RNA was reverse transcribed to complementary DNA (cDNA) in a reaction mix of Superscript II reverse transcriptase (Invitrogen, Paisley, UK), dNTP mix (Bioline, London, UK) and random primers (Promega, Southampton, UK) according to manufacturer instructions.

TaqMan PCR was performed using 5ng of input cDNA and Taqman PCR mastermix (Applied Biosystems, Warrington, UK) and human *THY-1* primer and probe set (Hs00174816_m1, Assay on demand, Applied Biosystems, Warrington, UK). Amplification and detection was performed using the ABI Prism 7700 Sequence Detection System (Applied Biosystems, Warrington, UK). *THY-1* mRNA was normalised to the geometric mean of C_T_ values for cytochrome c-1 (*CYC-1)* and topoisomerase DNA I (*TOP1)* as described previously [14]. Normalising genes were selected from a range of housekeeping genes using the Genorm protocol [17].

### Immunohistochemistry and TUNEL-labelling

Immunohistochemistry and TUNEL-labelling were used to assess the number of surviving RGCs in HORCs as described previously [14]. Briefly, HORCs were fixed in 4% formaldehyde for 24h and then cryopreserved in a 30% sucrose solution in PBS for a further 24h at 4°C. HORCs were mounted in Optimal Cutting Temperature compound (OCT) (Sakura Finetek, Zoeterwoude, Netherlands) and frozen at -80°C. 13μm retinal slices were taken using a Bright OTF 5000 cryostat (Bright Instruments, Huntingdon, UK) and mounted on 3’aminopropyl-triethoxyl silane (TESPA; Sigma-Aldrich, Poole, UK) coated glass slides. Assessment via Digital Vernier Caliper (Clarke, Essex, UK) ensured slices were taken at the centre of 4mm samples.

The primary antibody used was mouse monoclonal NeuN (1:200) (Chemicon International, Millipore, Watford, UK) and the secondary antibody was goat anti-mouse AlexaFluor 488 or 555 (1:1000) (Invitrogen, Paisley, UK). For the TUNEL assay (Promega, Southampton, UK), retinal slices were washed and immersed in TUNEL equilibration buffer for 10min, 18h after primary antibody binding. Slices were incubated in TUNEL reaction mixture for 1h at 35°C before stopping the reaction by immersion in standard citrate solution (SCS). After further washing, nuclei were stained with DAPI (1:100; Sigma-Aldrich, Poole, UK).

18 × 200μm sections from each HORC were counted in a masked fashion. The number of NeuN-labelled cells co-localising with DAPI were used as a measure of RGC number. NeuN positive cells which also stained positive for TUNEL were identified as apoptotic RGCs. It is important to note that there is no major staining of NeuN in the inner nuclear layer suggesting that NeuN does not label amacrine cells [14].

### Western blotting

Protein lysates were obtained from HORCs using Mammalian Protein Extract Reagent M-PER supplemented with Halt Phosphatase Inhibitor Cocktail, Protease Inhibitor Cocktail and 5mM EDTA (All from Thermo Scientific, Loughborough, UK) for 20min on ice followed by centrifugation at 13,000rpm for 5min. Protein concentration of each lysate was determined using a bicinchoninic acid (BCA) protein assay (Thermo Scientific, Loughborough, UK). Equal amounts of protein were loaded onto 10% SDS-PAGE gels and proteins separated by electrophoresis. Proteins were transferred to PVDF membrane (Perkin Elmer Life Sciences, Cambridge, UK) using a semi-dry transfer blotter (Bio-Rad Laboratories, Hemel Hempstead, UK). Membranes were blocked with PBS-T (0.1% Tween-20 in PBS, 5% fat-reduced milk), hybridized with primary antibody followed by incubation with secondary antibody (GE Healthcare, Buckinghamshire, UK). Bands were visualised using chemiluminescent ECL Plus Western Blot Detection reagent (GE Healthcare, Buckinghamshire, UK) and net band intensity determined (1D 3.5 software, Eastman Kodak, Rochester, NY). Primary antibodies (Cell Signaling Technology, Danvers, MA, USA) against phospho- and total p38, phospho- and total JNK were used at 1:250, 1:1000, 1:500 and 1:500 respectively.

### Statistical Analysis

Data shown is the mean ± standard error of the mean (S.E.M). Significance was determined using an unpaired Student’s t-test (GraphPad Prism version 6.0, San Diego, USA). Differences were considered significant at the p≤0.05 level. Groups were considered statistically similar if p≥0.2 (β=0.2) and p values are given throughout. Due to having only one chamber, pressure experiments were carried out independently using separate donors with appropriate same donor controls.

## Results

### Effect of increased hydrostatic pressure on RGC survival in HORCs

**Figure 3:**
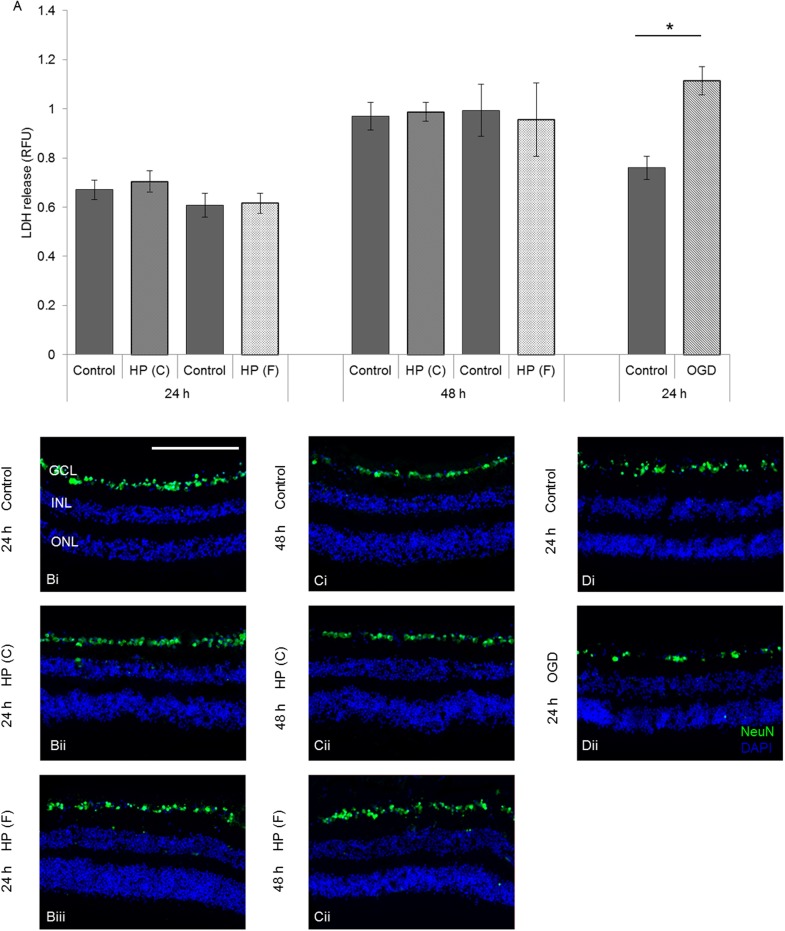
Elevated hydrostatic pressure (HP) did not cause necrotic cell death or loss of retinal structure in HORCs. (A) No increase in necrotic cell death, measured by released cytoplasmic LDH, was observed after constant (HP (C); 60mmHg) or fluctuating (HP (F); 10–100mmHg; 1cycle/min) pressure for 24 or 48h (HP(C) 60mmHg 24h—n = 20, p = 0.564; HP(C) 60mmHg 48h—n = 20, p = 0.907; HP(F) 10–100mmHg 24h—n = 8, p = 0.794; HP(F) 10–100mmHg 48h—n = 8; p = 0.838). A positive control of 3h OGD/21h control conditions led to a significant increase in released LDH compared to control conditions (n = 11; *p = 0.0001). (B-D) Representative immunofluorescence photomicrographs of HORCs; (B) 24h control (i) or pressure (ii, iii) exposure, (C) 48h control (i) or pressure (ii, iii) exposure and (D) 24h control (i) or 3h OGD/21h control conditions (ii). DAPI = blue, NeuN = green, GCL = ganglion cell layer, INL = inner nuclear layer, ONL = outer nuclear layer. Scale = 200μm.

There was no significant increase in released LDH as a result of either constant or fluctuating pressure at 24h (HP(C) 60mmHg—n = 20, p = 0.564; HP(F) 10–100mmHg 1 cycle/min—n = 8, p = 0.794) or 48h (HP(C) 60mmHg—n = 20, p = 0.907; HP(F) 10–100mmHg—n = 8, p = 0.838) compared with controls (Fig. 3A). As a positive control, simulated ischemia caused an approximate 50% increase in release of LDH into the culture medium at 24h, indicating that increased death of retinal cells had occurred under these conditions (n = 11, p = 0.0001; Fig. 3A). Retinal architecture was preserved in HORCs exposed to constant and fluctuating HP for 24 or 48h and OGD for 24h, with no observed differences between control and pressure groups or with simulated ischemia (Fig. 3B, C & D).

**Figure 4:**
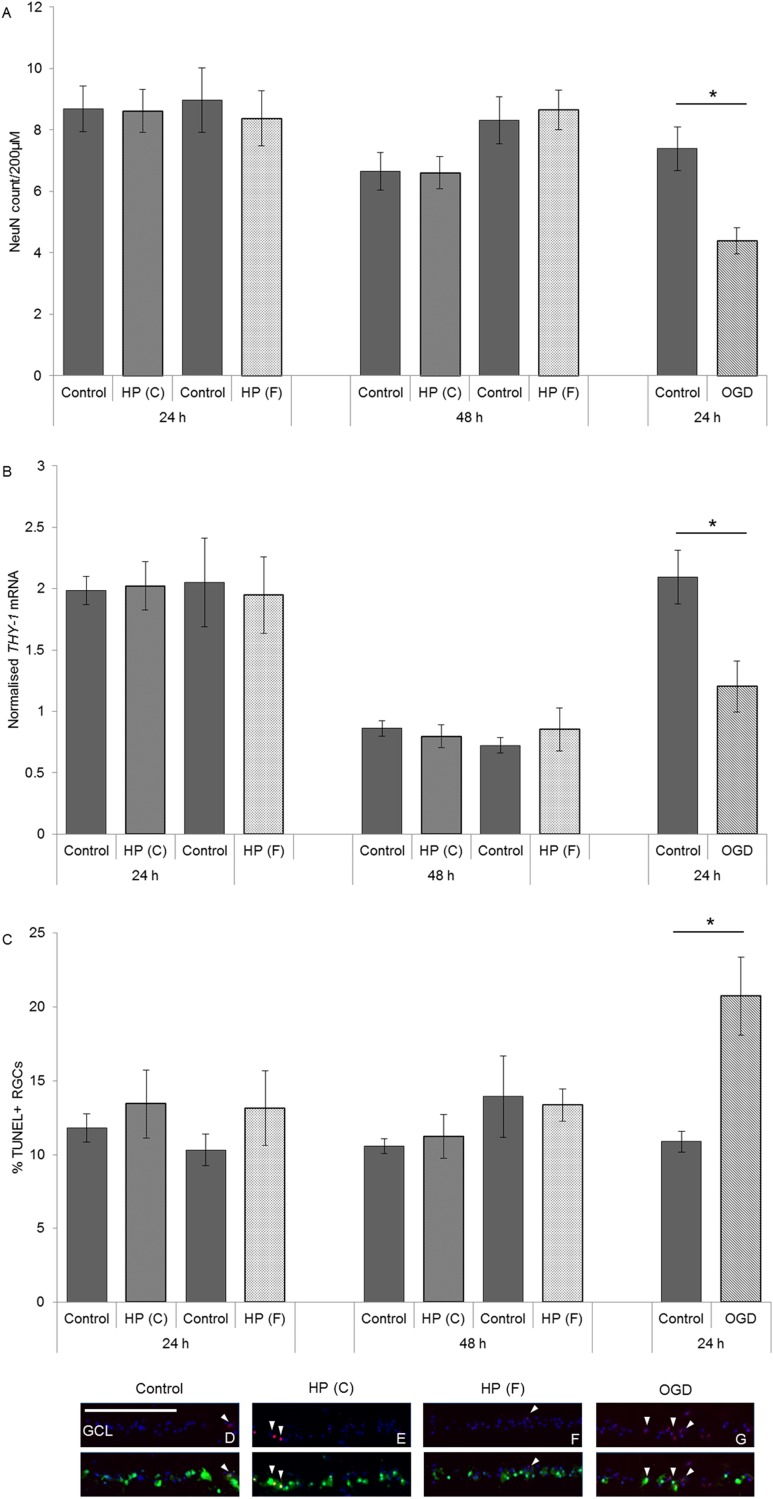
Elevated hydrostatic pressure did not decrease the expression of RGC specific markers in HORCs or cause RGC apoptosis. (A) Constant (HP(C); 60mmHg) or fluctuating (HP(F) 10–100mmHg; 1cycle/min) pressure did not decrease the number of NeuN-labelled RGCs at the 24 or 48h time-points (HP(C) 60mmHg 24h—n = 9, p = 0.947; HP(C) 60mmHg 48h—n = 9, p = 0.668; HP(F) 10–100mmHg 24h—n = 10, p = 0.955; (HP(F) 10–100mmHg 48h—n = 10; p = 0.733). A significant reduction in NeuN-labelled cells was observed following simulated ischemia (3h OGD/21h control conditions) (n = 9; *p = 0.002). (B) Elevated HP for 24 or 48h did not reduce *THY-1* mRNA expression compared to same time point controls (HP(C) 60mmHg 24h—n = 4, p = 0.878; HP(C) 60mmHg 48h—n = 4, p = 0.837; HP(F) 10–100mmHg 24h—n = 4, p = 0.584; HP(F) 10–100mmHg—n = 4; p = 0.516). A significant reduction in *THY-1* expression was caused by 3h OGD/21h control conditions (n = 8; *p = 0.010). (C-G) Apoptotic labelling in RGCs was low with no increase in the number of TUNEL+ NeuN-labelled cells at 24 or 48h after constant or fluctuating pressure compared to controls (HP(C) 60mmHg 24h—n = 4, p = 0.531; HP(C) 60mmHg 48h—n = 4, p = 0.349; HP(F) 10–100mmHg 24h—n = 4, p = 0.695; HP(F) 10–100mmHg—n = 4; p = 0.853). An increase in the proportion of apoptotic RGCs could be detected following 3h OGD/ 21h control conditions (n = 4; *p = 0.011). DAPI = blue, TUNEL = red, NeuN = green, GCL = ganglion cell layer. White arrows highlight TUNEL+ NeuN-labelled cells. Scale = 200μm.

Focussing more specifically on survival of RGCs in HORCs, NeuN labelling and *THY-1* mRNA expression were quantified (Fig. 4A&B). The numbers of NeuN-labelled neurons relative to controls did not change after exposure to either constant or fluctuating pressure for 24h (HP(C) 60mmHg—n = 9, p = 0.947; HP(F) 10–100mmHg—n = 10, p = 0.955) or 48h (HP(C) 60mmHg—n = 9, p = 0.668; HP(F) 10–100mmHg—n = 10, p = 0.733) (Fig. 4A). In addition, no significant change in the level of *THY-1* mRNA between control and pressure exposure at either time-point was observed with either pressure regime (HP(C) 60mmHg 24h—n = 4, p = 0.878; HP(C) 60mmHg 48h—n = 4, p = 0.837; HP(F) 10–100mmHg 24h—n = 4, p = 0.584; HP(F) 10–100mmHg 48h—n = 4; p = 0.516) (Fig. 4B). Simulated ischemia, however, caused an almost 50% reduction in the number of NeuN-labelled cells compared with controls (n = 9; p = 0.021; Fig. 4A) and a similar decrease in *THY-1* mRNA levels (n = 8; p = 0.010; Fig. 4B), indicating a reduction in RGC number.

Since it might be expected that decline in RGC number could occur later than 48h, but that apoptosis may have been initiated during this period, the number of TUNEL-positive NeuN-labelled cells was also assessed (Fig. 4C-G). No significant differences in the number of apoptotic RGCs were observed at either time-point using either pressure regime (HP(C) 60mmHg 24h—n = 4, p = 0.531; HP(C) 60mmHg 48h—n = 4, p = 0.349; HP(F) 10–100mmHg 24h—n = 4, p = 0.695; HP(F) 10–100mmHg 48h—n = 4; p = 0.853). OGD, on the other hand, caused an approximate doubling of the number of TUNEL-positive NeuN-positive cells at 24h (n = 4; p = 0.011) indicating that it was inducing significant apoptotic cell death by this time-point (Fig. 4G).

### Effect of hydrostatic pressure on p38 and JNK signalling

**Figure 5:**
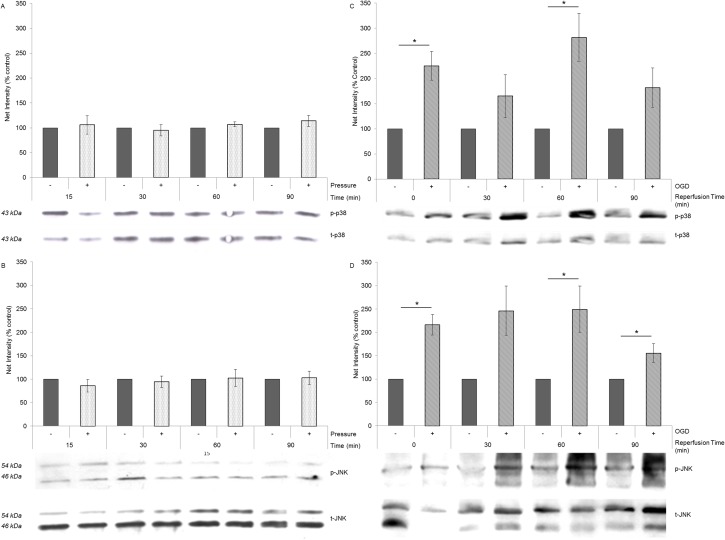
Elevated pressure did not activate p38 or JNK stress signalling pathways. Phosphorylation of (A) p38 and (B) JNK, relative to their total expression, did not significantly alter with fluctuating pressure in HORCs (n = 3; 15 min- p38 p = 0.769, JNK p = 0.354; 30 min—p38 p = 0.696, JNK p = 0.667; 60 min—p38 p = 0.232, JNK p = 0.891; 90min-p38 p = 0.273, JNK p = 0.833). Phosphorylation of (C) p38 and (D) JNK was observed immediately following 3h OGD (n = 3; 0 min—p38 p = 0.012, JNK p = 0.006), and in the during the following reperfusion period in control medium (n = 3; 60 min—p38 p = 0.019, JNK p = 0.039; 90 min—JNK p = 0.049). Results are expressed as a percentage of the untreated control. Representative blots are shown.

Investigation of the stress pathways p38 and JNK showed no increased activation (phosphorylation) in HORCs following exposure to fluctuating pressure (10–100mmHg; 1 cycle/min) at 15 min (n = 3; p38 p = 0.769; JNK p = 0.354), 30 min (n = 3; p38 p = 0.696; JNK p = 0.667), 60 min (n = 3; p38 p = 0.232; JNK p = 0.891) and 90min (n = 3; p38 p = 0.0.273; JNK p = 0.833) (Fig. 5A, B). HORCs exposed to simulated ischemia, however, showed a sustained increase in p38 and JNK phosphorylation compared to controls, with significant increases at the end of the OGD period (0 min; n = 3; p38 p = 0.012; JNK p = 0.006), at 60 min (n = 3; p38 p = 0.019; JNK p = 0.039) and 90 min (n = 3; JNK p = 0.049) post-OGD. Activation was therefore observed directly following the 3h OGD period and activation remained elevated at subsequent time points for 90min post-insult (Fig. 5C, D).

## Discussion

Although ocular hypertension has been identified as a major risk factor for glaucoma, precisely how raised IOP translates into loss of RGCs and consequent visual field deterioration is poorly understood. Several previous studies have suggested that increased HP can induce RGC death [9–12]. The aim of the present study was therefore to investigate whether similar pressure-induced loss of retinal cells could also be observed in the human retina using an explant (HORC) model.

Since we were using a custom-made pressure chamber, it was important to validate the system and consider any potential confounding factors. By using MFCs it was shown that HP could be accurately increased within the chamber and also be tightly regulated. Pressure increased to the target pressure within 30sec and was maintained within ±1mmHg. Using this system, we could be confident that no uncontrolled initial pressure surges were experienced by the tissue, such as could occur if the chamber were connected directly to a gas cylinder. Also using this system we could be confident that there was no movement of the tissue, either via fluid turbulence or movement of the underlying substrate. We were, in turn, confident that the tissue was exposed purely to raised HP and that we had not inadvertently introduced any mechanical distortion. We measured evaporation of medium from dishes in the chamber and found no difference at raised HPs compared to control dishes outside of the chamber, such that one would not anticipate any exposure to differing osmotic conditions. In addition, in design of the system we enabled a constant gas flow through the chamber, independent of pressure regulation, in order to mitigate against changes in gas composition (albeit very small due to the large volume of this chamber) as a result of tissue respiration. It does, however, have to be addressed, that some changes could not be mitigated against when using this design of chamber. Specifically, in chambers that increase HP by raising the gas pressure at a gas-liquid interface, the concentration of dissolved gases in the medium must be considered. Increasing pressure in the gas phase increases the partial pressure of each gas within this phase; this leads to a proportional increase in the concentration of dissolved gases, including O_2_, in the liquid phase (ie. the medium) as described by Henry’s Law. An increase in O_2_ was measured in the medium within our chamber (Fig. 2) in agreement with Henry’s Law. Therefore, any measured effects of raised HP in our system would have needed to take this increase in O_2_ into consideration. Raised partial pressure of CO_2_ would also occur, so it was also important to measure medium pH; this was not found to change significantly under the conditions of the experiment i.e. buffering of the medium was sufficient to compensate for the increased [CO_2_]. We were confident, therefore, that apart from an increase in [O_2_] as a result of Henry’s Law, that we had considered and addressed other potential confounding factors such that we would be able to interpret any changes seen in cell viability in terms of an effect of HP on the retinal cells.

Exposing the retinal explants to increased HP for up to 48h did not cause a reduction in RGC survival or induction of apoptosis in response to constant (60mmHg) or fluctuating pressure (10–100mmHg; 1 cycle/min). In contrast, as a positive control, we exposed HORCs to simulated ischemia which did cause significant loss of RGCs. Increased p38 and JNK phosphorylation has previously been described in animal models of glaucoma [18–21] and p38 or JNK pathway inhibition has been shown to protect RGCs following axotomy [22, 23] and ischemia [18]. In HORCs exposed to increased HP, no significant change in p38 and JNK phosphorylation was detected. HORCs subjected to simulated ischemia, however, showed increased p38 and JNK phosphorylation at early time-points, thus demonstrating the sensitivity of our model system.

To our knowledge, only one previous paper has investigated the effects of HP on retinal explants [12]. The research exposed rat retinal explants to raised HP and showed a loss of RGC viability, but only when the pressure was increased very rapidly (at approximately 8mmHg/s). A slower increase of approximately 3mmHg/s did not cause loss of viability. In our experiments, the rise was commensurate with the slower rate and therefore the results could be seen as consistent with this previous data. Whether we would see loss in viability with a greater rate of increase in HP could not be tested with our system, but it should be noted that such rapid changes in IOP would not be experienced in patients with glaucoma.

Other studies on the effects of raised HP have utilised isolated retinal cells, cultured on rigid, artificial substrates specifically glass and tissue culture plastic [9–11]. Although these cultures provide valuable information with regards to individual cell type responses, their usefulness as a model of the retina is limited due to lack of cell-matrix and cell-cell attachments and signalling between RGCs and the supporting glia and inner retinal cells. The fact that the cells are cultured on a rigid surface would exert extra forces when HP is raised which could impact RGC survival in this experimental system. Retinal explant models more closely reflect the cell organisation and interactions within the eye and although the HORC model does not maintain associations with the RPE, its basement membrane, the choroid and the sclera, the potential effects of HP on RGCs against their natural retinal substrate, the IPL and INL, are preserved. Neither model can therefore exactly replicate the *in vivo* environment of the eye. Differences between the outcomes using these experimental models could potentially be explained by these differences between the culture systems.

It should be remembered that HP only constitutes a small component of forces associated with elevated IOP, specifically, the transverse stress across the retina. In the eye *in vivo*, pressure is acting within a closed system and there is a differential pressure between the inside and outside of the eye. It can therefore be described in mechanical terms by modelling the effects of raising pressure within a closed vessel. Within a closed vessel, pressure has two mechanical effects: it directly causes a stress transversely through a section of the vessel wall (along a radial axis), but it also creates an in-plane tensile stress in the vessel wall, which resists stretching of the circumference. The latter stress is known as “hoop stress” and acts along the surface of a vessel wall in a circumferential direction. For a pressure vessel of radius 15mm and wall thickness of 1mm, the hoop stress would be 15 times greater than the transverse stress for a given increase in internal pressure. In the eye, the hoop stress would be experienced predominantly in the tissue with the highest tensile strength, specifically, the sclera. Associated strains would in turn be experienced in the adjacent tissues also in the orthogonal direction. The consequences of hoop stress as a result of increased IOP are therefore more likely to influence RGC survival compared to the transverse stress across the retina. Importantly, hoop stress would *not* be modelled in an experimental system where cells or tissue were cultured in dishes that are placed within a chamber where HP is raised.

In our experiments, it was found that applying HP to retinal explants did not result in RGC death or influence pathways associated with changes in survival. We would therefore suggest that the component of raised IOP that is modelled by increasing HP, i.e. the transverse stress across the retina that increases as IOP is raised, is not a direct contributor to RGC death. Certainly our results are consistent with the compelling argument that application of HP alone is not a surrogate for IOP in glaucoma [24, 25]. Investigators should therefore look more towards models that replicate strain/stress in ocular tissues as more appropriate models of the physical consequences of raised IOP. The rapidly expanding field of ocular biomechanics [26–30] will be critical in this respect and it certainly would be interesting to look further at the effects of hoop stress-associated strain, which could be modelled *in vitro* by orthogonal stretching of the retina. Further to this, it is clear that we need to learn more about the stress/strain relationships both between the retina and its adjacent structures and *within* the retina: could attachments of the RGCs and their relationship to, for example, the nerve fibre layer, cause stress in this region of the retina that makes the RGCs more susceptible to increased pressure than other retinal cells? Application of research from this important field will be critical in allowing the development of pathophysiologically relevant models to measure RGC death with respect to glaucoma.
